# Immunogenicity and safety of measles-mumps-rubella vaccine delivered by disposable-syringe jet injector in India: A randomized, parallel group, non-inferiority trial

**DOI:** 10.1016/j.vaccine.2018.01.006

**Published:** 2018-02-21

**Authors:** Ashish Bavdekar, Jitendra Oswal, Padmasani Venkat Ramanan, Chandrashekhar Aundhkar, P. Venugopal, Dhananjay Kapse, Tara Miller, Sarah McGray, Darin Zehrung, Prasad S. Kulkarni, SII MMR author group, D. Ramaganeshan, Amita Sapru, Anand Pandit, Anand Kawade, Sanjay Lalwani, Sonali Palkar, Nita Hanumante, Nandini Malshe, Vidya Krishna, S.Y. Ingale, Bhagwat Gunale, Amol Chaudhari, Laura Saganic, Courtney Jarrahian

**Affiliations:** e Andhra Medical College and King George Hospital, Visakhapatnam, India; a KEM Hospital Research Centre, Pune, India; f Shri Shirdi Sai Baba Hospital, Vadu, India; b Bharti Vidyapeeth Deemed University Medical College, Pune, India; c Sri Ramachandra Medical Centre, Chennai, India; d Krishna Institute of Medical Sciences, Karad, India; g Serum Institute of India Pvt. Ltd., Pune, India; i PATH, Seattle, USA; a KEM Hospital Research Centre, Pune, India; b Bharti Vidyapeeth Deemed University Medical College, Pune, India; c Sri Ramachandra Medical Centre, Chennai, India; d Krishna Institute of Medical Sciences, Karad, India; e Andhra Medical College and King George Hospital, Visakhapatnam, India; g Serum Institute of India Pvt. Ltd., Pune, India; h PharmaJet, Golden, USA; i PATH, Seattle, USA

**Keywords:** Disposable-syringe jet injector (DSJI), Needle-free, Vaccination, Measles-mumps-rubella (MMR) vaccine, Immunogenicity, Safety

## Abstract

**Background:**

We conducted a randomized, non-inferiority, clinical study of MMR vaccine by a disposable-syringe jet injector (DSJI) in toddlers in India in comparison with the conventional administration.

**Methods:**

MMR vaccine was administered subcutaneously by DSJI or needle-syringe (N-S) to toddlers (15–18 months) who had received a measles vaccine at 9 months. Seropositivity to measles, mumps, and rubella serum IgG antibodies was assessed 35 days after vaccination. Non-inferiority was concluded if the upper limit of the 95% CI for the difference in the percent of seropositive between groups was less than 10%. Solicited reactions were collected for 14 days after vaccination by using structured diaries.

**Results:**

In each study group, 170 subjects received MMR vaccine. On day 35, seropositivity for measles was 97.5% [95% CI (93.8%, 99.3%)] in the DSJI group and 98.7% [95% CI (95.5%, 99.8%)] in the N-S group; for mumps, 98.8% [95% CI (95.6%, 99.8%)] and 98.7% [95% CI (95.5%, 99.8%)]; and for rubella, 98.8% [95% CI (95.6%, 99.8%)] and 100% [95% CI (97.7%, 100.0%)]; none of the differences were significant. The day 35 post-vaccination GMTs in DSJI and N-S groups were measles: 5.48 IU/ml [95% CI (3.71, 8.11)] and 5.94 IU/ml [95% CI (3.92, 9.01)], mumps: 3.83 ISR [95% CI (3.53, 4.14)] and 3.66 ISR [95% CI (3.39, 3.95)] and rubella: 95.27 IU/ml [95% CI (70.39, 128.95)] and 107.06 IU/ml [95% CI (79.02, 145.06)]; none of the differences were significant.

The DSJI group reported 173 solicited local reactions and the N-S group reported 112; most were mild grade. Of the total of 156 solicited systemic adverse events, most were mild, and incidence between the two groups was similar.

**Conclusions:**

MMR vaccination via DSJI is as immunogenic as vaccination by N-S. Safety profile of DSJI method is similar to N-S except for injection site reactions which are more with DSJI and are well-tolerated.

**Registration**

US National Institutes of Health clinical trials identifier – NCT02253407.

Clinical trial registry of India identifier – CTRI/2013/05/003702

## Highlights

• We compared MMR vaccine administration by disposable-syringe jet injector (DSJI) and needle and syringe (N-S).
• The study was conducted in 340 toddlers who had received a measles vaccine at 9 months.
• On day 35, seropositivity for all three viruses was more than 97% in both the groups.
• Reactogenicity by both methods was comparable.
• MMR vaccination via DSJI is as immunogenic and safe as vaccination by N-S.

## 1 Introduction

Worldwide, measles caused 89,780 deaths in 2016, mostly among children under age five [1]. Rubella is generally a mild viral infection in children, but in pregnant women it may cause fetal death or severe congenital defects [2]. As a result, the World Health Organization (WHO) recommends measles and rubella vaccines for all the children in the world [3]. In 2012, several global agencies led by the WHO set the goal of eliminating measles and rubella in at least five WHO regions by 2020 [4]. Currently, global measles immunization coverage is at 85%, but, to achieve elimination, at least 95% coverage with two doses of vaccine is required [5]. Though measles vaccine is used universally, in many developing countries, vaccines against mumps and rubella are not used in immunization programs.

Measles-mumps-rubella (MMR) vaccine generally is administered as a subcutaneous injection with needle and syringe (N-S). However, vaccine delivery with needles can cause needle-stick injuries and cross-infection, and it also creates dangerous sharps waste in communities. N-S delivery also complicates the logistics of immunization campaigns, since the requirement for proper sharps disposal can limit their reach and coverage. An alternative to N-S for vaccine delivery is the jet injector, a device that creates a fine stream of pressurized liquid that penetrates the skin to deposit vaccine without using a needle [6].

Disposable-syringe jet injectors (DSJIs) that use a sterile, single-dose, disposable syringe for each patient were introduced in the 1990s, and a number of models have been approved in the United States and Europe for different uses, including vaccinations [6].

The risks associated with DSJI use include failure to deliver the correct dose; pain, bleeding, or swelling at the injection site; and user error in positioning the injector to deliver the dose to the correct layer of tissue—however, most of these risks also apply to vaccination by N-S [7].

Vaccination by jet injection has been shown to induce immunity similar to that provided by N-S injection and to have a similar safety profile for a number of vaccines, including typhoid, cholera, smallpox, hepatitis A and B, influenza, whole cell pertussis-diphtheria-tetanus, polio, yellow fever, and tetanus. [6] A previous study comparing a DSJI with N-S for administering MMR vaccine several years ago met the requirement for rubella but failed to demonstrate non-inferiority of the DSJI to N-S for the measles and mumps vaccines [8]. However, that study used a different jet injector than the one used in the present study as well as a vaccine from a different manufacturer.

We conducted a phase IV, randomized, observer-blind, non-inferiority, parallel-group, multicentric clinical study of MMR vaccination in infants in India to compare immunogenicity and safety of the vaccine when administered by a DSJI to administration by conventional N-S method. A result of non-inferiority for the DSJI would support use of vaccination with a jet injector, offering a needle-free alternative for country immunization programs.

## 2 Methods and materials

The study sponsor was the Serum Institute of India Pvt. Ltd. (SIIPL, Pune, India). DiagnoSearch Life Sciences (Mumbai, India) was delegated by the sponsor for site monitoring, project management, clinical data management, and statistical analysis of the data. Approvals were obtained from the Drug Controller General of India, the institutional ethics committees of all study centers, and the Western Institutional Review Board in the United States. The study was carried out in accordance with the Declaration of Helsinki and the ICH Harmonized Tripartite Guideline for GCP (E6) 1996; the GCP Guidelines in India; and the Ethical Guidelines for Biomedical Research on Human Subjects, issued by Indian Council of Medical Research in 2006.

### 2.1 Vaccine

MMR vaccine (SIIPL, India) was used in the study. It is presented as a single-dose of lyophilized vaccine and is provided with a sterile diluent (0.5 ml of water for injection) in a separate container. The vaccine is reconstituted by adding the diluent to the vial containing the lyophilized pellet. A single dose of 0.5 ml contains live attenuated strains of Edmonston-Zagreb measles virus (not less than 1000 CCID50), Leningrad-Zagreb mumps virus (not less than 5000 CCID50), and Wistar RA 27/3 rubella virus (not less than 1000 CCID50). The same batch of the vaccine (MMR batch 013N4017A expiry May2016 and diluent batch 064Q40330Z expiry April 2016) was used throughout the study. It was stored at 2–8 °C. The dose was 0.5 ml by both delivery methods.

### 2.2 Injection devices

**Fig. 1:**
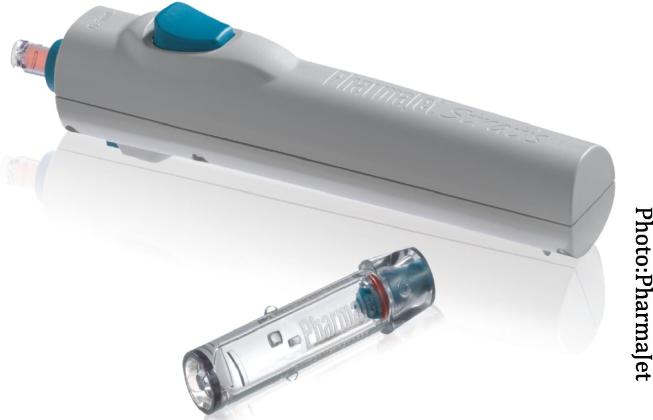
Stratis SC/IM (0.5 ml fixed dose).

The investigational product for this study was the MMR vaccine administered subcutaneously by the Stratis DSJI (PharmaJet, Golden, Colorado, USA) (Fig. 1). This device is licensed for use in the United States and in the European Economic Area; it is also prequalified by WHO [9], [10], [11]. The Stratis needle‐free injection system delivers 0.5 ml fluid volumes either intramuscularly or subcutaneously by means of a precise narrow fluid stream, which penetrates the skin in about a 1/10th of a second and delivers the medicine or vaccine into the body. Energy to propel the fluid is supplied by a hand-held, spring-powered injector, designed to be reused a minimum of 20,000 times. A disposable syringe containing medicine or vaccine is attached to the injector and placed in contact with the patient’s skin. The fluid is then expelled through a very small orifice in the face of the syringe.[10] The batch numbers for the Stratis devices used in the study were 25854275 and 23436455. The reference product was the same MMR vaccine administered subcutaneously via N-S.

### 2.3 Study populations and settings

The study was conducted at six sites across India from September 2014 through December 2015. Eligible participants were healthy children aged 15–18 months who had received a measles vaccine at 9 months of age. Children with a past history of measles, mumps, or rubella infection; significant abnormality; any neoplasm or blood disorder; or a history of allergy to any of the vaccine components and those who had previously received the MMR vaccine were not eligible. After written informed consent from their parents, 5 ml blood was drawn for immunogenicity testing from eligible subjects, and the MMR vaccine was administered subcutaneously in the anterolateral aspect of the thigh on day 0 by either of the two techniques.

Parents were issued subject diaries and educated to fill in the solicited adverse reactions as well as other reactions for 14 days and asked to return to the study site for follow up visits on day 14 (2nd visit) and day 35 (3rd visit).

### 2.4 Randomization and blinding

A block randomization scheme was used to allocate eligible subjects in a 1:1 ratio to receive MMR vaccine either by DSJI or N-S. Each block consisted of six subjects. The randomization list was generated using SAS® statistical software version 9.2 in SAS Enterprise Guide 4.2 (SAS Institute, Cary, North Carolina, USA). The list of randomization numbers and the group allocations covered with scratch labels were provided to all sites. Subjects were allocated to groups by scratching the label corresponding to the randomization number in the list by the vaccinator, just before vaccine administration. Investigator site personnel—except for staff administering the vaccine—and laboratory staff were not aware of the allocation.

### 2.5 Immunogenicity evaluations

A blood sample was collected from each subject at baseline and on day 35 after vaccination. Paired serum samples were tested using ELISA IgG kits (Trinity Biotech, Bray, Ireland) at Quest Diagnostics (Gurgaon, India). Seropositivity for each vaccine component was defined as IgG antibody titers ≥1.10 immune status ratio (ISR). For measles and rubella, antibody titers were converted from ISR to IU/ml per instructions in the ELISA kits. For mumps, the ISR values were used. Geometric mean titers (GMTs) were calculated for the secondary endpoint.

### 2.6 Safety evaluations

At each visit, subjects were examined by the study physician and a history was taken for adverse events (AEs) and concomitant medications. AEs were coded using the Medical Dictionary for Regulatory Activities (MedDRA), Version 18.0 [12]. Parent diaries for solicited local and systemic AEs occurring over 14 days post-vaccination were transcribed into the case report form. All solicited events were recorded for maximum severity and relatedness to treatment. The solicited local reactions were pain, redness, swelling, or bruising. The solicited systemic AEs were fever, rash, parotitis, lymphadenopathy, and loss of appetite. Unsolicited events and serious events were also collected from subjects throughout their entire participation in the study. All adverse events were categorized into mild, moderate or severe based on pre-defined severity criteria. As the investigational product was the combination of delivery device with vaccine, it was not possible to categorize AEs by component (vaccine or device related).

### 2.7 Delivery evaluations

Data on injection quality were recorded immediately post injection. The absence or presence and severity of injection site trauma was recorded and residual wetness remaining on the skin was measured using blotting paper. The absence or presence of crying and duration of cry was also noted.

### 2.8 Statistical analyses

All statistical analyses were performed using SAS statistical software, version 9.2. Sample size was determined under the assumption that 90%of individuals in the control group would become seropositive, and that 10% would withdraw from the study. A total of 340 subjects were enrolled to provide at least 80% power to rule out a difference in percentage seropositivity of greater than 10% between groups, using a one-sided significance level of 0.025 for each vaccine component.

The intention-to-treat (ITT) population was used for baseline and safety analysis. The per-protocol (PP) population (those with no major protocol deviations impacting immunogenicity analysis and who completed all three visits with evaluable blood samples on day 0 and day 35) was used for immunogenicity analysis.

Percent seropositivity was calculated as the percentage of subjects for whom the day 35 post-vaccination titer was ≥1.10 ISR. The percentage and 95% confidence interval (CI) of seropositivity for measles, mumps, and rubella between the two groups was compared using the Farrington and Manning method [13]. Non-inferiority was concluded if the upper limit of the 95% CI for the difference in the percent seropositive between groups was less than 10%.

The GMTs of antibodies between DSJI and N-S groups were compared between groups using a two sample *t*-test. Pre- and post-dose seropositivity and GMTs within each group were compared using McNemar’s chi-square test and a paired *t*-test, respectively. Safety endpoints were the proportion of solicited local and systemic AEs, unsolicited AEs, and serious AEs (SAEs) throughout the study. The intention-to-treat population was used for safety analyses. Further details are provided in the supplementary material.

A post hoc immunogenicity analysis was performed for measles seropositivity at day 35 in subjects who were measles seronegative at baseline. The percentage and 95% CI were compared between groups using the Farrington and Manning method.

## 3 Results

**Fig. 2:**
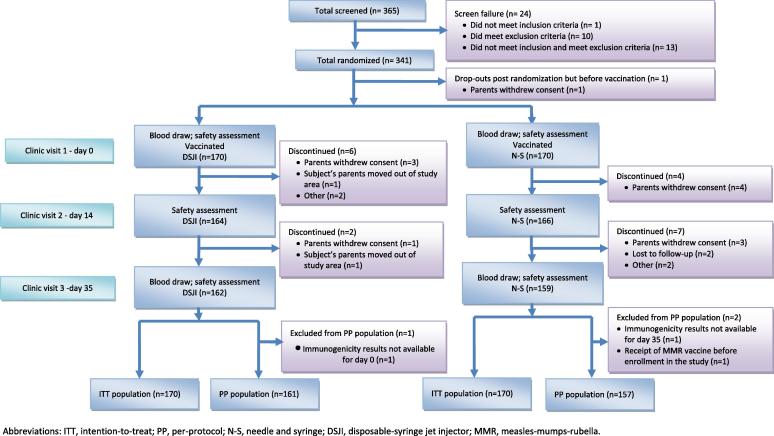
Study flowchart.

A total of 365 subjects were screened and 341 eligible subjects were randomized. The parents of one subject withdrew consent after randomization but before vaccination; thus, a total of 340 subjects received study vaccine, 170 in each group (Fig. 2). At baseline, the DSJI and N-S groups were similar in age, weight, and height; however, there were more males in the DSJI group (Table 1, p = .039).

**Table 1:** Baseline characteristics: Intention-to-treat population.#

Characteristic*	DSJI (n = 170)	N-S (n = 170)
*Age (months)*		
Mean (SD)	16.4 (1.1)	16.3 (1.1)

*Height (cm)*		
Mean (SD)	77.5 (3.3)	77.9 (2.7)

*Weight (kg)*		
Mean (SD)	9.5 (1.2)	9.4 (1.1)

*Gender*^		
Male, n (%)	97 (57.1%)	77 (45.3%)
Female, n (%)	73 (42.9%)	93 (54.7%)

# The intention-to-treat population is all participants who received the study vaccine.

* All subjects were of Indian ethnicity.

^ Numbers of males and females in the study groups were significantly different (p = .039, Fisher’s exact test).

### 3.1 Immunogenicity results

At baseline, seropositivity rates were similar between both the groups for all three antigens (Table 2). On day 35, seropositivity rates in the DSJI and N-S groups were 97.5% [95% CI (93.8%, 99.3%)] and 98.7% [95% CI (95.5%, 99.8%)] for measles; 98.8% [95% CI (95.6%, 99.8%)] and 98.7% [95% CI (95.5%, 99.8%)] for mumps; and 98.8% [95% CI (95.6%, 99.8%)] and 100% [95% CI (97.7%, 100.0%)] for rubella. All seropositivity rates were comparable between the two groups. In addition, there was a significant rise in the proportions of seropositive subjects from baseline to day 35 within each group for all three components (p = .0001, chi-square test, data not shown). In subjects who were seronegative for measles at baseline, more than 95% were seropositive at day 35 in both groups, and the difference between groups was not significant (data not shown).

**Table 2:** Seropositivity* at day 0 and at day 35 after vaccination in the per-protocol population.β

Vaccine component		Statistic			Day 35		
		DSJI (n = 161)	N-S (n = 157)	Two-sided p-value by Fisher’s Exact Test	DSJI (n = 161)	N-S (n = 157)	Difference in percentage^
Measles	Seropositive subjects (%)	100 (62.1)	107 (68.2)	–	157 (97.5)	155 (98.7)	1.2
	2-Sided 95% CI	(54.1, 69.6)	(60.3, 75.4)	0.2902	(93.8, 99.3)	(95.5, 99.8)	(−4.0, 6.4)

Mumps	Seropositive subjects (%)	13 (8.1)	10 (6.4)	–	159 (98.8)	155 (98.7)	−0.1
	2-Sided 95% CI	(4.4, 13.4)	(3.1, 11.4)	0.6665	(95.6, 99.8)	(95.5, 99.8)	(−5.0, 4.9)

Rubella	Seropositive subjects (%)	7 (4.3)	6 (3.8)	–	159 (98.8)	157 (100.0)	1.2
	2-Sided 95% CI	(1.8, 8.8)	(1.4, 8.1)	1.0000	(95.6, 99.8)	(97.7, 100.0)	(−3.7, 6.2)

* IgG antibody titers were determined by ELISA (Trinity Biotech) for each vaccine component. Seropositivity was defined as IgG antibody titers ≥1.10 immune status ratio (ISR), according to the levels given in the Trinity Biotech kit. For measles and rubella, antibody titers were converted from ISR to IU/ml per instructions in the Trinity Biotech kits. For mumps, the ISR values were used. All samples were tested in duplicate and the mean of the two values was used. Repeat testing was performed on samples with equivocal results.

β The per-protocol population consisted of all subjects who had no major protocol violations and who completed all three clinic visits, with evaluable blood samples at day 0 and day 35.

^ Two-sided 95% CI is estimated for the difference between proportions using the Farrington and Manning method. The upper limit of the two-sided 95% CI for the percentage of seropositivity for all vaccine components was less than 10%; thus, the seropositivity of the MMR DSJI group was non-inferior to that of the MMR N-S group.

GMTs were not significantly different between the two groups at baseline (Table 3). At day 35 after vaccination, in the DSJI group, GMTs were 5.48 IU/ml, 3.83 ISR, and 95.27 IU/ml for measles, mumps, and rubella, respectively. As for the comparable values in the N-S group at day 35, GMTs were 5.94 IU/ml, 3.66 ISR, and 107.06 IU/ml (p > .05). There was a significant rise in GMTs for all three antigens from baseline to day 35 in both groups (Table 3).

**Table 3:** Geometric mean titers of anti-measles, anti-mumps, and anti-rubella antibody concentrations* on day 0 and day 35 after vaccination in the per-protocol population.

Vaccine component	Statistic	Day 0		Day 35	
		DSJI (n = 161)	N-S (n = 157)	DSJI (n = 161)	N-S (n = 157)
Measles (IU/ml)	Geometric mean titer (GMT)	0.19	0.17	5.48	5.94
	Two-sided 95% CI	(0.15, 0.26)	(0.13, 0.21)	(3.71, 8.11)	(3.92, 9.01)
	*p*-Value (between groupsα)	.4571		.7813	
	p-Value (within groupβ)			<.0001	<.0001

Mumps (ISR)	GMT	0.29	0.29	3.83	3.66
	Two-sided 95% CI	(0.25, 0.32)	(0.25, 0.33)	(3.53, 4.14)	(3.39, 3.95)
	p-Value (between groupsα)	.9821		.4293	
	p-Value (within groupβ)			<.0001	<.0001

Rubella (IU/ml)	GMT	3.25	3.04	95.27	107.06
	Two-sided 95% CI	(2.73, 3.86)	(2.77, 3.34)	(70.39, 128.95)	(79.02, 145.06)
	p-Value (between groupsα)	.5150		.5914	
	p-Value (within groupβ)			<.0001	<.0001

* IgG antibody titers were determined by ELISA (Trinity Biotech) for each vaccine component.

α For comparing GMTs between the two study groups, the two-sample *t*-test was used.

β For comparing GMTs within each group between day 0 and day 35, the paired *t*-test was used. The p-value is shown in the day 35 columns.

For subjects who were **seronegative** for measles at baseline, GMTs for measles were 0.04 IU/ml [95% CI (0.04, 0.05)] at Day 0 and 2.92 IU/ml [95% CI (1.45, 5.89)] at Day 35 in DSJI group; while in N-S group it was 0.05 IU/ml [95% CI (0.04, 0.05)] at Day 0 and 3.59 IU/ml [95% CI (1.78, 7.23)] at Day 35. For subjects who were **seropositive** for measles at baseline, GMTs for measles were 0.49 IU/ml [95% CI (0.35, 0.69)] at Day 0 and 8.04 IU/ml [95% CI (5.10, 12.69)] at Day 35 in DSJI group; while in N-S group it was 0.31 IU/ml [95% CI (0.24, 0.40)] at Day 0 and 7.51 IU/ml [95% CI (4.48, 12.61)] at Day 35.

### 3.2 Safety results

A total of 285 solicited local reactions were reported, with173 in the DSJI group and 112 in the N-S group, a statistically significant difference (Table 4). Pain was the most frequently reported reaction in both groups (44.7% in DSJI group and 35.3% in N-S group). In the DSJI group, 54.7% of subjects had mild intensity local reactions and 8.8% of subjects had moderate intensity local reactions; in the N-S group, the proportion was 40.6% and 5.9% respectively. Only one subject (0.59%) had severe intensity local reaction i.e. pain in the DSJI group. No severe local reaction was reported in N-S group. All reactions resolved without sequelae.

**Table 4:** Solicited local reactions and systemic adverse events by study groups, intention-to-treat population.

DSJI (n = 170)				N-S (n = 170)			p-Valueα
	No. subjects	No. events	% subjects (95% CI)	No. subjects	No. events	% subjects (95% CI)	
*Local adverse events*							
Pain	76	81	44.7 (37.1, 52.5)	60	61	35.3 (28.1, 43.0)	.096
Redness	40	42	23.5 (17.4, 30.6)	22	22	12.9 (8.3, 18.9)	.016
Swelling	47	48	27.6 (21.1, 35.0)	27	27	15.9 (10.7, 22.3)	.012
Bruising	2	2	1.2 (0.1, 4.2)	2	2	1.2 (0.1, 4.2)	1.00
At least one local reaction	97*	173 (Mild: 155; Moderate: 16; Severe: 1) Severity for one local reaction i.e. redness is missing	57.1 (49.3, 64.6)	75*	112 (Mild: 99; Moderate: 13; Severe: Nil)	44.1 (36.5, 51.9)	.02

*Systemic adverse events*							
Loss of appetite	34	44	20.0 (14.3, 26.8)	29	35	17.1 (11.7, 23.6)	.58
Fever	19	19	11.2 (6.9, 16.9)	20	20	11.8 (7.3, 17.6)	1.00
Rash	13	15	7.6 (4.1, 12.7)	12	13	7.1 (3.7, 12.0)	1.00
Lymphaden-opathy	4	5	2.4 (0.6, 56.0)	2	2	1.2 (0.1, 4.2)	0.25
Parotitis	3	3	1.8 (0.4, 5.1)	0	0	0 (0.0, 2.2)	0.68
At least one event	51*	86 (Mild: 68; Moderate: 14; Sever: 4)	30.0 (23.2, 37.5)	46*	70 (Mild: 56; Moderate: 11; Severe: 3)	(20.5, 34.4)	0.63

α p-Value for number of subjects calculated using Fisher’s exact test.

* Total number of subjects with at least one local reaction or systemic adverse event is less than the sum of the numbers for that column because some subjects experienced more than one event.

Out of 156 solicited systemic AEs, 86 were reported in the DSJI group and 70 in the N-S group. The most commonly reported were loss of appetite, fever, and rash (Table 4). In the DSJI group, loss of appetite was reported in 20% of subjects, fever in 11.2%, and rash in 7.6%, compared with 17.1%, 11.8%, and 7.1%, respectively, in the N-S group. The incidence of solicited systemic AEs between the two groups was similar.

A total of 371 unsolicited AEs (including SAEs) were reported in 185 subjects across both the groups (178 in DSJI group and 193 in N-S group—data not shown). Nine events (four injection site haemorrhage; one lymphadenopathy; one parotitis; and three upper respiratory tract infections) in DSJI group and seven events (one injection site haemorrhage; one injection site induration; four upper respiratory tract infections; and one vomiting) in N-S group were related to investigational or reference product (investigational product is the combination of delivery method and vaccine; causality is not attributable to the separate components). Incidence of unsolicited AEs was comparable between the two groups, and most of these AEs were of mild intensity. Four SAEs were reported during the study, two in each of the study groups, with the seriousness criteria of hospitalization. All were unrelated to the study vaccination, and none of the subjects was discontinued from the study. Further details are in the supplementary material. All local and systemic AEs reported during the study period resolved without sequelae.

Most injections for both groups resulted in a single drop of residual fluid at the site after injection: for the DSJI group, this proportion was 78%, and for the N-S group, it was 64%. In the DSJI group, 15% had a completely dry site, and in the N-S group, the proportion was 35% (see supplementary material).

## 4 Discussion and conclusions

The seropositivity following MMR vaccine administered using the DSJI was non-inferior to that for vaccine administered via N-S for all three components of the vaccine with a non-inferiority margin of 10%; thus, the primary efficacy endpoint of the study was met. Also, differences in Day 35 post-vaccination GMTs between two groups for each component of vaccine were not statistically significant. A booster effect was seen for measles with MMR vaccination in both the groups in subjects who were seropositive at baseline. There was more increase in seropositivity from baseline to Day 35 post-vaccination for mumps and rubella in N-S group as compared to DSJI group, and the opposite was seen for measles. Similarly, there was more rise in GMTs from baseline to Day 35 post-vaccination for measles and rubella in N-S group as compared to DSJI group, and the opposite was seen for mumps. However, these apparent small differences were not statistically compared as they were not part of statistical analysis plan. Injection site reactions were more in DSJI group as compared to N-S group and this difference between two groups was statistically significant. However all injection site reactions resolved without any sequelae. Similarly, in a previous study of influenza vaccination, higher frequency of local injection site reactions were reported with DSJI than with the use of needle and syringe [14]. Systemic adverse reactions were comparable between the two study groups. Nine unsolicited adverse events (four injection site haemorrhage; one lymphadenopathy; one parotitis; and three upper respiratory tract infections) in DSJI group and seven unsolicited adverse events (one injection site haemorrhage; one injection site induration; four upper respiratory tract infections; and one vomiting) in N-S group were related to investigational or reference product (investigational product is the combination of delivery method and vaccine; causality is not attributable to the separate components). All reported systemic adverse events were consistent with typical MMR vaccination adverse events.

Since all subjects had received a measles vaccination at 9 months of age, around 65% were measles seropositive at the beginning of the study. However, for mumps and rubella, the baseline seropositivity was less than 10%. After vaccination, the proportions of seropositives in both the groups increased significantly, to a level of 98–100% for all three antigens, indicating that administration with the DSJI results in immunogenicity similar to that after injection with N-S.

As noted earlier, jet injectors have worked well with many licensed vaccines, with the exception of a study of MMR vaccine conducted in Brazil that failed to demonstrate non-inferiority of the DSJI to N-S for measles and mumps vaccines [6], [8]. The device used in that study was discontinued by the manufacturer and replaced with the Stratis device used in the current study. One hypothesis for the outcome of the Brazil study was that the pressures and shear forces generated during jet injection might have affected the viability of the live viruses in MMR vaccine; however, subsequent laboratory studies found that this was not the case [15]. Another possibility was that vaccine left on the surface of the skin might have contributed to the reduced immunogenicity after DSJI delivery. The different manufacturers’ MMR vaccines also could have contributed to the difference in results. Thus, ours is the first study that demonstrates that MMR vaccine can be given by a jet injector with equivalent immunogenicity as that with conventional N-S. Also, MMR vaccination by jet injector is as safe as vaccination by N-S except for injection site reactions, in particular redness and swelling, which are more with DSJI. The cause of the increased injection site reactions with DSJI is not proven, but may be due to the mechanism of action of the DSJI, which deposits residual amounts of vaccine at each layer of the skin as it penetrates to the correct delivery depth.

Limitations of this study include the lack of masking of the study participants and their parents to the method of vaccination and unequal gender distribution in the two study groups. An unequal gender distribution was purely a random occurrence. The use of block randomization can introduce bias, particularly with smaller block sizes.

To conclude, subcutaneous MMR vaccination via DSJI is as immunogenic as vaccination by N-S. MMR vaccination by DSJI demonstrates a clinically acceptable safety profile and is similar to vaccination by N-S except for injection site reactions which are more with DSJI and are well-tolerated. Results of this study support use of the DSJI for MMR vaccination and provide information for regulatory authorities, immunization program managers, and clinicians who make decisions about safe clinical practice standards. Using the DSJI can reduce the risks of needle-stick injuries and the burden of sharps waste disposal, which can streamline logistics and contribute to improved coverage in low-resource settings, helping to reach the goal of preventing these diseases and their serious sequelae.

## Funding

Bill & Melinda Gates Foundation and Serum Institute of India Pvt. Ltd.

## References

[1] World Health Organization. Measles fact sheet; 2017. http://www.who.int/mediacentre/factsheets/fs286/en/ [accessed 7.12.17].

[2] World Health Organization. Rubella fact sheet; 2017. http://www.who.int/mediacentre/factsheets/fs367/en/ [accessed 7.12.17].

[3] World Health Organization. Recommended routine immunizations for children; 2017. http://www.who.int/immunization/policy/Immunization_routine_table 2.pdf?ua=1 [accessed 7.12.17].

[4] World Health Organization (WHO). Global measles and rubella strategic plan 2012–2020. Geneva (Switzerland): WHO; 2012. http://www.who.int/immunization/documents/control/ISBN_978_92_4_150339_6/en/ [accessed 20.10.16].

[5] World Health Organization (WHO). Global vaccine action plan. Monitoring, evaluation & accountability. Secretariat annual report. Geneva (Switzerland): WHO; 2015. http://who.int/immunization/global_vaccine_action_plan/gvap_secretariat_report_2015.pdf [accessed 20.10.16].

[6] Zehrung D, Jarrahian C. Technologies to improve immunization. In: Bloom B, Lambert P-H, editors. The vaccine book, second ed. Cambridge (MA): Academic Press; 2016.

[7] John Clements C., Larsen Gordon, Jodar Luis (2004). Technologies that make administration of vaccines safer. Vaccine.

[8] de Menezes Martins R, Curran B., Maia Mde L., Ribeiro Md., Camacho L.A., da Silva Freire M (2015). Immunogenicity and safety of measles-mumps-rubella vaccine delivered by disposable-syringe jet injector in healthy Brazilian infants: a randomized non-inferiority study. Contemp Clin Trials.

[9] Special 510(k) Premarket Notification, PharmaJet®, Inc., PharmaJet® stratis needle-free injection system; July 27, 2011, http://www.accessdata.fda.gov/cdrh_docs/pdf11/k111517.pdf [accessed 20.10.16].

[10] PharmaJet. Stratis IM/SC; 2016. http://pharmajet.com/product/ [accessed 20.10.16].

[11] PharmaJet. PharmaJet’s Stratis® needle-free injector receives WHO PQS certification as a pre-qualified delivery device for vaccine administration [blog post]; February 20, 2013. http://pharmajet.com/pharmajets-stratis-needle-free-injector-receives-pqs-certification-pre-qualified-delivery-device-vaccine-administration/ [accessed 20.10.16].

[12] International Committee on Harmonization. Medical dictionary for regulatory activities (MedDRA); 2016. http://www.meddra.org/. What’s new MedDRA version 18.0.2015, http://www.meddra.org/sites/default/files/guidance/file/whatsnew_18_0_english.pdf [accessed 20.10.16].

[13] Farrington C.P., Manning G. (1990). Test statistics and sample size formulae for comparative binomial trials with null hypothesis of non-zero risk difference or non-unity relative risk. Stat Med.

[14] McAllister Linda, Anderson Jonathan, Werth Kristen, et al. Needle-free jet injection for administration of influenza vaccine: a randomised non-inferiority trial; 2014 [published online May 30, 2014], 10.1016/S0140-6736(14)60524-9. 10.1016/S0140-6736(14)60524-9 24881803

[15] Coughlin M.M., Collins M., Saxon G., Jarrahian C., Zehrung D., Cappello C. (2015). Effect of jet injection on infectivity of measles, mumps, and rubella vaccine in a bench model. Vaccine.

