# Supplementary material for: Immunogenicity and safety of measles-mumps-rubella vaccine delivered by disposable-syringe jet injector in India: A randomized, parallel group, non-inferiority trial
Source: Vaccine. 2018 Feb 21;36(9):1220–6. doi: 10.1016/j.vaccine.2018.01.006 (PMC5818644; doi:10.1016/j.vaccine.2018.01.006)
Supplement: Supplementary data 1 [file mmc1.docx]

**Supplementary material**

**Materials and Methods**

**Vaccine.** The vaccine used in this study was an approved MMR vaccine from SIIPL, administered subcutaneously. Single dose vials were used. Each reconstituted 0.5-ml dose contained the following active ingredients:

- Live attenuated measles virus, Edmonston-Zagreb strain, not less than 1,000 CCID50
- Live attenuated mumps virus, Leningrad-Zagreb strain, not less than 5,000 CCID50
- Live attenuated rubella virus, Wistar RA 27/3 strain, not less than 1,000 CCID50

Stabilizers included partially hydrolyzed gelatin, sorbitol, L-histidine, L-alanine, tricine, L-arginine hydrochloride, and lactalbumin hydrolysate. The diluent was water-for-injection.

**Names and locations of the centers where the study was conducted.**

- Department of Paediatrics, Andhra Medical College, King George Hospital, Maharanipet, Visakhapatnam, Andhra Pradesh
- Krishna Institute of Medical Sciences, Deemed University, Karad, Maharashtra
- Shirdi Sai Baba Rural Hospital, KEM Hospital Research Centre, Vadu Budruk, Maharashtra
- Department of Paediatrics, KEM Hospital Research Centre, Pune, Maharashtra
- Department of Paediatrics, Bharati Vidyapeeth Deemed University Medical College and Hospital, Pune, Maharashtra
- Department of Paediatrics, Sri Ramachandra Medical Centre, Chennai, Tamil Nadu

All data from all subjects enrolled at the six sites were used for the final analysis. No formal comparisons were made for vaccination by center or geographic region.

**Clinic visits.** There were three clinic visits: at the first on day 0 included a baseline blood draw, subcutaneous administration of MMR vaccine in the anterolateral aspect of the thigh, and monitoring of adverse events (AEs) for one hour following vaccination. Parents were given a diary for recording injection site reactions such as redness, swelling, and pain; and for systemic reactions such as fever, loss of appetite, or parotitis. At the second visit on day 14, subjects had a general physical examination and a medical history was taken. The final clinic visit occurred within days 35 to 49 and included the post-vaccination blood draw and evaluation of delayed local reactions or systemic AEs.

**Table A. Reactions to be solicited and assessment of severity**

| **Local reactions (at the injection site)** | **Severity grade** |
| --- | --- |
| Pain at injection site |  |
| Present, but leg movement not affected | 1 (mild) |
| Discomfort, interfered with or limits leg movement | 2 (moderate) |
| Disabling, unable to move leg | 3 (severe) |
| Redness, swelling, or bruising |  |
| Longest diameter: <20mm | 1 (mild) |
| Longest diameter: ≥20mm to ≤50mm | 2 (moderate) |
| Longest diameter: >50mm | 3 (severe) |
| **Systemic reactions** | **Severity grade** |
| Fever (axillary temperature measured once daily even in the absence of signs) | |
| 38°C to <39°C | 1 (mild) |
| 39°C to <40°C | 2 (moderate) |
| ≥40°C | 3 (severe) |
| **General observations (local and systemic, including rash, parotitis, lymphadenopathy, and loss of appetite)** | **Severity grade** |
| Symptom noted, but no disruption of normal daily activities; slightly bothersome; relieved with or without symptomatic treatment | 1 (mild) |
| Symptoms sufficient to reduce or affect normal daily activity to some degree; bothersome; interferes with activities; only partially relieved with symptomatic treatment | 2 (moderate) |
| Symptoms sufficient to reduce or affect normal daily activity considerably (prevents regular activities for at least 24 hours);not relieved with symptomatic treatment; would cause parent to seek medical advice | 3 (severe) |

**Statistical analyses**

For baseline clinical and demographic variables, categorical variables were described by number and percentage of subjects randomized for each study group, and compared using ANOVA (age, height, weight) or Fisher’s Exact Test (gender). Descriptive statistics (mean, standard deviation, median, and range) were calculated for continuous variables and compared across study groups using analysis of variance.

For the primary efficacy analysis, the proportions of individuals seroconverting on day 35 for measles, mumps, and rubella were calculated and compared using the Farrington and Manning method, based on two-sided 95% CIs estimated for the difference between means, under the assumption that the concentration was normally distributed. Thus, seropositivity was calculated as the percentage of subjects for whom the day 35 post-vaccination titer was ≥ 1.10 ISR. If the lower limit of the two-sided 95% CI for the proportion of seropositivity was less than 0.1, we would conclude that the seropositivity of the MMR DSJI group was non-inferior to that of the MMR N-S group. The primary efficacy analysis was performed on the per-protocol (PP) population, defined as all subjects who had no major protocol violations and who completed all three clinic visits, with evaluable blood samples at day 0 and day 35.

Comparison of seropositivity within each group at day 0 and day 35 postvaccination was performed using McNemar’s chi-square test. The GMT of anti-measles, anti-mumps, and anti-rubella antibody titers were compared between the two groups by the two-sample t-test. Comparison of the GMT of anti‑measles, anti-mumps, and anti-rubella antibody titers within each group at day 0 and day 35 postvaccination was performed using the paired t-test.

**The data safety committee** appointed by the sponsor was responsible for oversight of the trial conduct. Members of the committee included a physician as an independent safety evaluator, the principal investigators, the study statistician, and representatives of the sponsor.

**Results**

**Safety results**

Safety analyses were performed on the intention-to-treat population, which was defined as all participants who received at least one dose of study vaccine.

**Table B. Solicited systemic adverse reactions by intensity in the intention-to-treat population**

| **Systemic adverse reaction** | **DSJI (n = 170) n (%), X** | | | **N-S (n = 170) n (%), X** | | |
| --- | --- | --- | --- | --- | --- | --- |
|  | **Mild** | **Moderate** | **Severe** | **Mild** | **Moderate** | **Severe** |
| Loss of appetite | 26 (15.3%), 33 | 8 (4.7%), 8 | 3 (1.8%), 3 | 22 (12.9%), 25 | 7 (4.1%), 8 | 2 (1.2%), 2 |
| Fever | 16 (9.4%), 16 | 3 (1.8%), 3 | 0 | 19 (11.2%), 19 | 1 (0.6%), 1 | 0 |
| Rash | 10 (5.9%), 12 | 2 (1.2%), 2 | 1 (0.6%), 1 | 9 (5.3%), 10 | 2 (1.2%), 2 | 1 (0.6%), 1 |
| Lymphadenopathy | 4 (2.4%), 5 | 0 | 0 | 2 (1.2%), 2 | 0 | 0 |
| Parotitis | 2 (1.2%), 2 | 1 (0.6%), 1 | 0 | 0 | 0 | 0 |
| Any | 45 (26.4%), 68 | 11 (6.5%), 14 | 3 (1.8%), 4 | 42 (24.7%), 56 | 8 (4.7%), 11 | 2 (1.2%), 3 |

n = number of subjects with at least one AE (subjects counted only once)

X = number of events (a subject may be counted more than once)

**Table C. Summary of serious adverse events by study groups**

|  | **DSJI (n = 170)** | | **N-S (n = 170)** | |
| --- | --- | --- | --- | --- |
| **Serious adverse event (SAE)** | **n (%), X** | **95% CI** | **n (%), X** | **95% CI** |
| Total number of SAEs | 2 (1.18%), 2 | (0.1%, 4.2%) | 2 (1.18%), 2 | (0.1%, 4.2%) |
| Gastroenteritis | 1 (0.59%), 1 |  | 2 (1.18%), 2 |  |
| Lower respiratory tract infection | 1 (0.59%), 1 |  | 0 |  |

n = number of subjects with at least one SAE (subjects counted only once)

X = number of events (a subject may be counted more than once)

**Unsolicited adverse events.** Across both groups, 371 unsolicited AEs were reported—178 in the DSJI group and 193 in the N-S group—in a total of 185 subjects. Nine events (4 injection site hemorrhage, 1 lymphadenopathy, 1 parotitis, and 3 upper respiratory tract infection) in the DSJI group and seven events (1 injection site hemorrhage, 1 injection site induration, 4 upper respiratory tract infection, and 1 vomiting) in the N-S group were judged related to the study vaccine administered by the DSJI or N-S. All these events were mild in intensity, and all resolved without sequelae, except for three events of anemia and one event of lymphadenopathy that were ongoing at the time of the last study visit. All of these were transferred to clinical care.

**Table D. Summary of injection observations post-vaccination**

| **Observation** | **Details** | **DSJI (n = 170)**  **n (%)** | **N-S (n = 170)**  **n (%)** |
| --- | --- | --- | --- |
| Fluid at injection site | No fluid; site was dry | 25 (14.7%) | 60 (35.3%) |
|  | Skin was damp at injection site | 4 (2.4%) | 2 (1.2%) |
|  | Single drop of fluid at injection site | 133 (78.2%) | 108 (63.5%) |
|  | Fluid dripped from injection site | 3 (1.8%) | 0 |
|  | Fluid oozed from injection site | 2 (1.2%) | 0 |
|  | Completely wet shot | 3 (1.8%) | 0 |
| Blood at injection site | None | 68 (40.0%) | 102 (60.0%) |
|  | Drop | 98 (57.6%) | 67 (39.4%) |
|  | Flow | 4 (2.4%) | 1 (0.6%) |
| Infant crying | None | 30 (17.6%) | 26 (15.3%) |
|  | Already crying before injection* | 71 (41.8%) | 83 (48.8%) |
|  | Brief cry | 69 (40.6%) | 61 (35.9%) |
|  | Inconsolable cry | 0 | 0 |
| Erythema | Yes | 2 (1.2%) | 0 |
|  | No | 168 (98.8%) | 170 (100.0%) |
| Laceration at injection site | Yes | 1 (0.6%) | 0 |
|  | No | 169 (99.4%) | 170 (100.0%) |

* The high proportion of infants crying before injection in both groups may be attributable to the fact that they had blood drawn from a wrist vein—a painful procedure—shortly before the vaccination, in the same room.
